# 4‐1BB and cytokines trigger human NK, **γδ** T, and CD8^+^ T cell proliferation and activation, but are not required for their effector functions

**DOI:** 10.1002/iid3.749

**Published:** 2022-12-15

**Authors:** Laurent Vidard

**Affiliations:** ^1^ Department of Immuno‐Oncology Sanofi Vitry‐sur‐Seine France

**Keywords:** 4‐1BB costimulation, activation, CD8^+^ T cells, IL‐15, IL‐21, natural killer cells, proliferation, γδ T cells

## Abstract

**Introduction:**

This study was designed to compare the costimulatory molecules and cytokines required to trigger the proliferation and activation of natural killer (NK), γδ T, and CD8^+^ T cells, and gain in‐depth insight into the mechanisms shifting tolerance to immunity.

**Methods:**

K562‐derived artificial antigen‐presenting cells (aAPCs); that is, K562 forced to express CD86 and 4‐1BBL costimulatory receptors, in the presence of cytokines, were used to mimic dendritic cells (DCs) and provide signals to support the proliferation and activation of NK, γδ T, and CD8^+^ T cells.

**Results:**

Three signals are required to trigger optimal proliferation in MART‐1‐specific CD8^+^ T cells: activation of T‐cell receptors (TCRs) by the major histocompatibility complex (MHC) I/peptide complexes (signal 1); 4‐1BB engagement (signal 2); and IL‐15 and IL‐21 receptor co‐signaling (signal 3). NK and γδ T cell proliferation also require three signals, but the precise nature of signal 1 involving cell‐to‐cell contact was not determined. Once they become effectors, only signal 1 determines the sensitivity or resistance of the target cells to cytolysis by killer lymphocytes. When freshly purified, none had effector functions, except the NK cells, which could be activated by CD16 engagement.

**Conclusions:**

Therefore, lymphocytes committed to kill are produced as inactive precursors, and the license to kill is delivered by three signals, allowing for extensive proliferation and effector function acquisition. This data challenges the paradigm of anergy and supports the danger signal theory originally proposed by Polly Matzinger, which states that killer cells are tolerant by default, thereby protecting the mammalian body from autoimmunity.

## Graphical abstract

Three signals are required to trigger primary NK, gamma/delta T, and CD8 + T cell proliferation and activation. When freshly purified, none had effector functions, except the NK cells, which could be activated by CD16 engagement. Therefore, lymphocytes committed to kill are produced as inactive precursors, and the license to kill is delivered by three signals, allowing for extensive proliferation and effector function acquisition.

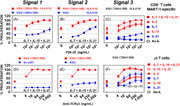

## 1 INTRODUCTION

Antigen and pathogen recognition by immune cells relies on specific receptors which are either germline‐encoded or somatically rearranged. It is generally accepted that immune cells can be classified as either innate or adaptive, except for NK and γδ T cells which express both types of receptors. Since 1959, when Sir Frank MacFarlane Burnet^1^ first described the clonal selection theory for acquired immunity, adaptive immunity has been a major focus in immunology, with innate immunity considered to be a more primitive form. Both B and T cells use sophisticated receptors to recognize a large repertoire of antigens, as a result of the somatic recombination of variables, diversity, and the joining (V(D)J) of gene segments.^2^ However, in 1989, Charlie Janeway went against the common opinion and proposed that innate cells bear receptors that allow for the recognition of pathogen‐associated molecular patterns that are not found in the host; that is, pattern recognition receptors (PRRs).^3^ The first PRR cloned was TLR4, the lipopolysaccharide receptor,^4^, ^5^, ^6^ and since then, many more have been identified.^7^

Autoimmunity was accepted by the immunology community in the 1960s (see reference^8^ for an outstanding review). The detection of autoantibodies in normal individuals has since led immunologists to elaborate and focus on the paradigm of discrimination between self‐ and non‐self‐antigens. A two‐signal model was initially proposed, in which, antigen receptor engagement alone (signal 1) inhibited lymphocytes unless accompanied by a second signal leading to lymphocyte activation.^9^, ^10^ In 1980, the term anergy was used by Nossal and Pike^11^ to describe the unresponsive state of lymphocytes induced by antigens in the absence of costimulation (signal 2). In parallel, a specialized T cell population has been proposed to control autoreactive lymphocytes.^12^, ^13^ Therefore, anergy and suppression were identified as the two arms (i.e., anergy and suppression performed coexistent mechanisms for the maintenance of peripheral T cell tolerance) controlling either activation (immunity) or unresponsiveness (tolerance) of anti‐non‐self and self‐reactive lymphocytes, respectively (see reference^14^ for an elegant review on anergy and^13^ on suppression).

DCs are recognized as the most potent professional APCs. However, as DCs have a costly, complicated, and time‐consuming preparation process, K562‐derived aAPCs have been engineered to mimic and replace them. K562‐derived aAPCs have been successfully used to amplify antigen‐specific CD8^+^ T,^15^ γδ T,^16^, ^17^ and NK^18^, ^19^ cells. While DCs were originally described as strong and specific T cell activators, as they present antigenic peptides in complex with MHC molecules, they were later also shown to be involved in processes that led to the proliferation and activation of NK^20^, ^21^, ^22^ and γδ T^23^, ^24^ cells. Consequently, DCs not only express MHC I and II molecules, but also a large set of ligands for costimulatory and adhesion molecules, and produce many different cytokines. The scarcity of DCs and their heterogeneity further add complexity to the mechanisms and pathways involved in the activation of the three major immune killer cells. Furthermore, in literature, the factors and processes that trigger proliferation and activation, especially for NK cells, remain ambiguous. Clarification of the mechanisms that lead to immunity—that is, killing of tumor cells—could also improve our understanding of the mechanisms that lead to tolerance.

For the first time, a comparison was performed here between NK, γδ T, and CD8^+^ T cells in parallel to assess the signals required to trigger both their proliferation and effector function acquisition. The aAPCS allow for the activation of resting lymphocytes and the expansion of numerous NK, γδ T, and antigen‐specific CD8^+^ T cells. The methodology used in adoptive cell therapies can be exploited for in vitro purposes; for example, to improve our understanding of the mechanisms driving NK, γδ T, and CD8^+^ T cell proliferation and activation at the molecular level. The proliferation and activation of NK, γδ T, and CD8^+^ T cells requires three signals: (i) cell‐to‐cell contact (signal 1), (ii) engagement of the costimulatory receptor 4‐1BB (signal 2), and (iii) signaling via the common γ‐chain (γc) cytokine receptors in the presence of IL‐15 and IL‐21 (signal 3). IL‐15 is one of the SOS signals sent by innate immune cells upon danger recognition,^25^ to both adaptive and other innate immune cells. Once lymphocytes differentiate into effector cells, only signal 1 determines the sensitivity or resistance of the target cells to effector killer cell cytolysis.

## 2 MATERIALS AND METHODS

### 2.1 Cell lines

Melanoma SK‐MEL‐5 (HTB‐70; ATCC) and A375 (CRL‐1619; ATCC) cell lines were maintained in DMEM (21969‐035; Life Technologies) supplemented with 10% heat‐inactivated fetal bovine serum (FBS; 10500056; Life Technologies) and 2 mM l‐glutamine (25030‐024; Life Technologies). The melanoma Malme‐3M (HTB‐64; ATCC) cell line was maintained in IMDM (31980‐032; Life Technologies) supplemented with 20% heat‐inactivated FBS and 2 mM l‐glutamine. Multiple myeloma U‐266 (ACC 9; DSMZ), chronic myelogenous leukemia (CML) K562 (CLL‐243; ATCC), acute lymphoblastic leukemia (ALL) NALM‐6 (available internally) cell line, Burkitt's lymphoma RAMOS (CRL‐1596; ATCC), and Daudi (CLL‐213; ATCC) cell lines were all maintained in RPMI 1640 (31870‐025; Life Technologies) supplemented with 10% heat‐inactivated FBS and 2 mM l‐glutamine. When NK, γδ T, or MART‐1‐specific HLA‐A2‐restricted CD8^+^ T cells were cocultured with the K562 tumor cell line or aAPC derivatives, the previous medium was also supplemented with 50 µM β2‐mercaptoethanol (31350‐010; Life Technologies), 50 µg/mL gentamicin (G1522; Sigma) and, unless otherwise mentioned, 100 U/ml (25 ng/ml) of IL‐7 (581908; BioLegend), 50 U/ml (150 ng/ml) of IL‐15 (570308; BioLegend), and 100 U/ml (50 ng/ml) of IL‐21 (571208; BioLegend). To maintain the CD86/4‐1BBL‐ and/or HLA‐A*02‐engineered aAPCs, the previous medium was supplemented with 1 µg/ml of puromycin (P9620; Sigma) and/or 270 µg/ml of zeocin (R25001, Invitrogen).

### 2.2 Reagents

The following reagents and antibodies were used: anti‐4‐1BB antibody (clone 4B4‐1, BioLegend, 309811; clone BBK‐2, LS‐C88297, LifeSpan BioSciences Inc; urelumab, produced internally), anti‐CD28 antibody (clone CD28.2, 16‐0289‐85; eBiosciences), anti‐NKp30 (clone P30‐15, 325204; BioLegend), anti‐NKp46 (clone 9E2, 331904; BioLegend), anti‐CD16 antibody (clone 3G8, 302050; BioLegend), anti‐CD20 (rituximab, hcd20‐mab1; Invivogen), anti‐CD3 (clone OKT3, 317326; BioLegend), anti‐TCR γδ (clone B1, 331236; BioLegend), MART‐1 (26‐35) peptide (EAAGIGILTV) (H‐4102.0005; Bachem), and NY‐ESO‐1 (157‐164‐V) peptide (SLLMWITQV; custom peptide synthesis, 96% pure; Bachem).

### 2.3 Engineering aAPCs

The cloning strategy and CD86/4‐1BBL construct used in this study were described previously.^26^ HLA‐A*02 (HLA‐A*02010101) and MART‐1 (MLANA, NM_005511) were synthesized by GeneArt as transcription units flanked by one CMV promoter and one polyadenylation recognition motif. When necessary, silent mutations were introduced to remove unwanted restriction sites before synthesis. A schematic representation of the constructs is shown in Supporting Information: Figure S1.

K562 cells were nucleofected according to the manufacturer's instructions and as described previously.^26^ The cells were then incubated for 3 d at 37°C in an incubator (5% CO_2_ and 95% relative humidity). Antibiotics (Supporting Information: Figure S1) were added to the culture medium, and cells were maintained under selection for 3–4 weeks. The expression of the transgene by the different K562‐derived aAPCS is depicted in Supporting Information: Figures S2 and S3.

### 2.4 Staining procedure and flow cytometry

Flow cytometry experiments were performed according to the guidelines published by Cossarizza et al.^27^ The cells were stained as previously described.^26^ The following antibodies and reagents were used, in accordance with the manufacturer's instructions: anti‐CD3 BV510 (clone UCHT‐12, 563109; BD), anti‐CD56 BV421 (clone NCAM16, 562751; BD), anti‐CD16 BV711 (clone 3G8, 302044; BioLegend), anti‐CD69 BUV737 (clone FN50, 564439; BD), anti‐NKG2D PE‐CF594 (clone 1D11, 562498; BioLegend), anti‐CD4 BUV395 (clone RPA‐T4, 564724; BD), anti‐TCRαβ PE‐Cy7 (clone IP26, 306720; BioLegend), anti‐TCRγδ FITC (clone 11F2, 347903; BD), anti‐CD8α BV650 (clone RPA‐T8, 301042; BioLegend), anti‐CD8b BV650 (clone 2ST8.5H7, 742393; BD), Anti‐CD86 PE‐Cy7 (clone IT2.2, 305422; BioLegend), anti‐4‐1BBL APC (clone 5F4, 311506; BioLegend), anti‐4‐1BBL PE (clone 5F4, 311504; BioLegend), anti‐HLA‐A2 PE (Clone BB7.2, 343306; BioLegend), anti‐HLA‐A/B/C PE (Clone W6/32, 311406; BioLegend), Dextramer HLA‐A*0201/ELAGIGILTV‐PE (WB2162‐PE; Immudex), and Dextramer HLA‐A*0201/SLLMWITQV‐PE (WB3247‐PE; Immudex).

The cytometer (BD LSRFortessa X20 cell analyzer) settings were calibrated and adjusted each day after running the cytometer setup and tracking (CST) beads and using the FACS Diva application settings (BD). OneComp eBeads (01‐1111‐42; eBioscience) were used as compensation controls. In all experiments, lymphocytes were defined based on the FSC × SSC gate. To identify viable and single cells, FSC‐A by APC‐Cy7‐A and FSC‐A by FSC‐H gates were used. Data were analyzed using FlowJo^TM^ V10.6.1 software.

### 2.5 Western blot

The cells were washed three times in DPBS (14190‐094; Life Technologies) and pellets were lysed in IPB buffer (0.5% NP40 [NP40S‐100ML; Sigma], 0.5% *N*‐nonanoyl‐N‐methylglucamine [N1138‐500MG; Sigma], 150 mM NaCl [55150‐1L; Sigma], 5 mM EDTA [03690‐100 ML; Sigma], 50 mM TRIS [T1699‐100ML; Sigma], 5 mM iodoacetamide [I1149‐5G; Sigma], and 2 mM PMSF [78830‐5G; Sigma]). The proteins in the cell extracts were quantified using the Micro BCA™ Protein Assay Kit (23235; Pierce) according to the manufacturer's instructions. Samples (20 µg of protein in 20 µL) were loaded on a precast NuPAGE™ Novex™ 4–12% Bis‐Tris gel (NP0322PK2; Invitrogen) and run at 120 V for 75–120 min. Proteins were transferred onto a nitrocellulose membrane (IB301001; Invitrogen) using an iBlot™ 2 Gel Transfer Device (IB21001; Invitrogen) according to the manufacturer's instructions. Nitrocellulose membranes were saturated in TBS (T5912‐L; Sigma) with 0.1% Tween‐20 (P7949; Sigma) and 5% nonfat milk (Merck, 115363) overnight at 4°C. The following antibodies and reagents were used according to the manufacturer's instructions: anti‐MART‐1 (clone M2‐7C10, MA5‐15237; Invitrogen), anti‐NY‐ESO‐1 (Clone E978, 35‐6200; Invitrogen), anti‐mouse IgG HRP polyclonal antibody (W402; Promega), anti‐GAPDH HRP antibody (Clone 14C10, 3683; Cell Signaling), and western lightning ECL Pro (NEL120001EA; PerkinElmer). Data acquisition was performed using ImageQuant™ LAS‐4000 (GE Healthcare), and the data were analyzed using Multi Gauge software (Fujifilm, V3.0).

### 2.6 NK, γδ T, and CD8^+^ T cell expansion

Buffy coats from healthy donors were provided by the Etablissement Français du Sang (EFS) after obtaining written informed consent. NK cells were purified using the Human NK Cell Isolation Kit (130‐092‐657; Miltenyi), γδ T cells were purified using the Human γδ T Cell Isolation Kit (19255C; Stemcell), and CD8^+^ T cells were purified using the Human CD8^+^ T Cell Isolation Kit (17953C; Stemcell), and all kits were used in accordance with the manufacturer's instructions. MART‐1‐specific and NY‐ESO‐1‐specific HLA‐A*02‐restricted CD8^+^ T cells were isolated from HLA‐A*02 PBMCs (PB009C‐3; HemaCare) using either MHC‐I‐*Strep* HLA‐A*0201 MART‐1 (ELAGIGILTV) (6‐7007‐002; IBA) or MHC‐I‐*Strep* HLA‐A*0201 NY‐ESO‐1 (SLLMWITQV) (6‐7013‐002; IBA), and *Strep*‐Tactin Magnetic Nanobeads (6‐5500‐005; IBA) according to the manufacturer's instructions. The purity of the NK, γδ T, and CD8^+^ T cells were evaluated using flow cytometry and was in the range of 85%–95% (Supporting Information: Figure S4), and viability was ≥95%.

A total of 400,000 freshly purified NK cells were cocultured with 8 × 10^5^ 50‐Gy x‐irradiated aAPCs (K562 CD86/4‐1BBL) in 3 ml of culture medium, and unless otherwise mentioned, 50 U/ml (150 ng/ml) of IL‐15 and 100 U/ml (50 ng/ml) of IL‐21. When necessary, the cells were further diluted with a cytokine‐containing medium. After one week, 30–60 ml of the NK cells was obtained. Cell numbers and viability were monitored weekly. Every other week, the cells were either allowed to rest in a cytokine‐containing medium (10^7^ NK cells in 30 ml of medium) or were restimulated with the addition of 50‐Gy x‐irradiated aAPCs and cytokines (5 × 10^6^ NK cells + 10^7^ aAPCs in 30 ml of medium). γδ T cells were expanded using the same procedure and by adding an anti‐TCR γδ antibody (clone B1, 100 ng/ml).

MART‐1‐specific HLA‐A*02‐restricted CD8^+^ T cells were purified from 200 million HLA‐A*02 PBMCs using the HLA‐A*02/P26‐35 heptamer. Based on the frequency of MART‐1‐specific HLA‐A*02‐restricted CD8^+^ T cells in the normal donors^28^, ^29^ which is approximately 0.1%, and that 50% of the T cells were present in the starting PBMCs (data not shown), it was estimated that 100,000 MART‐1‐specific HLA‐A*02‐restricted CD8^+^ T cells were isolated. T cells were mixed with either 50‐Gy x‐irradiated K562 CD86/4‐1BBL HLA‐A*02/MART‐1 or K562 CD86/4‐1BBL HLA‐A*02 in the presence of soluble MART‐1 peptide P26‐35 (3 µg/ml) for 3 days in the presence of IL‐21. The cultures were then incubated in the presence of IL‐7 + IL‐15 + IL‐21 for 12 d, and a cytokine‐containing medium was added when necessary. From Day 14, the cell numbers and viability were monitored weekly. Every other week, the cells were either allowed to rest in a cytokine‐containing medium (IL‐7 + IL‐15 + IL‐21; 10^7^ T cells in 30 mL of medium) or were restimulated with the addition of 50‐Gy x‐irradiated aAPCs (either K562 CD86/4‐1BBL HLA‐A*02/MART‐1 or K562 CD86/4‐1BBL HLA‐A*02 plus 3 µg/mL of peptide P26‐35) and cytokines (5 × 10^6^ T cells + 10^7^ aAPCs in 30 mL of medium). NY‐ESO‐1‐specific HLA‐A*02‐restricted CD8^+^ T cells were expanded using the same procedure (K562 CD86/4‐1BBL HLA‐A*02 plus 3 µg/ml of peptide P157‐164‐V).

### 2.7 Cell proliferation

Proliferation was monitored using cell counts and trypan blue dye exclusion with a Vi‐Cell XR cell counter (Beckman Coulter) or a WST‐1 colorimetric assay (11644807001; Roche) and a Victor X5 Light Plate Reader (2030‐0050; PerkinElmer) with a 450 nm filter.

For the WST‐1 assay, freshly purified NK and γδ T cells were seeded at 4,000 cells per well and the MART‐1‐specific HLA‐A*02‐restricted CD8^+^ T cells were seeded at 10,000 cells per well in 96‐well flat‐bottom plates in duplicate in complete RPMI medium. A total of 8,000 (for NK and γδ T cells) or 20,000 (MART‐1‐specific HLA‐A*02‐restricted CD8^+^ T cells) x‐irradiated (50‐Gy) aAPCs were added, and unless otherwise stated, antibodies, cytokines, peptides, or medium were added in 100 µL volumes to adjust the final volume of the culture to 200 µL. Cultures were incubated for 7 d at 37°C and 5% CO_2_. After 1 week, 20 µL of the WST‐1 reagent was added to each well, and the plates were incubated for an additional 4 h at 37°C and 5% CO_2_. The absorbance was measured at 450 nm using a Victor X5 light plate reader. Data were normalized by calculating the percentage of proliferation using the formula: (test OD 450 nm/maximum OD 450 nm) × 100.

### 2.8 Calcein release assay

The calcein release assay was performed as previously described.^26^ Briefly, two‐fold serial dilutions of the effector cells were made in 96‐well flat‐bottom plates in duplicate. A total of 20,000 prelabeled target cells were added to each well. Volumes were adjusted to 200 µL per well by adding either 50 µL of medium or antibody solution in RPMI complete medium.

After 4 h at 37°C and 5% CO_2_, 100 µL of the supernatant was collected and transferred into black 96‐well plates (Greiner, 655076). Fluorescence was recorded on a Victor X5 Light Plate Reader with excitation and emission wavelengths of 485 and 535 nm, respectively. Maximum and spontaneous release controls were set up in duplicate using 1% Triton X‐100 and RPMI complete medium, respectively. Specific lysis was calculated using the formula [(test release−spontaneous release)/(maximum release−spontaneous release)] × 100.

### 2.9 Statistical analysis

**Figure 1:**
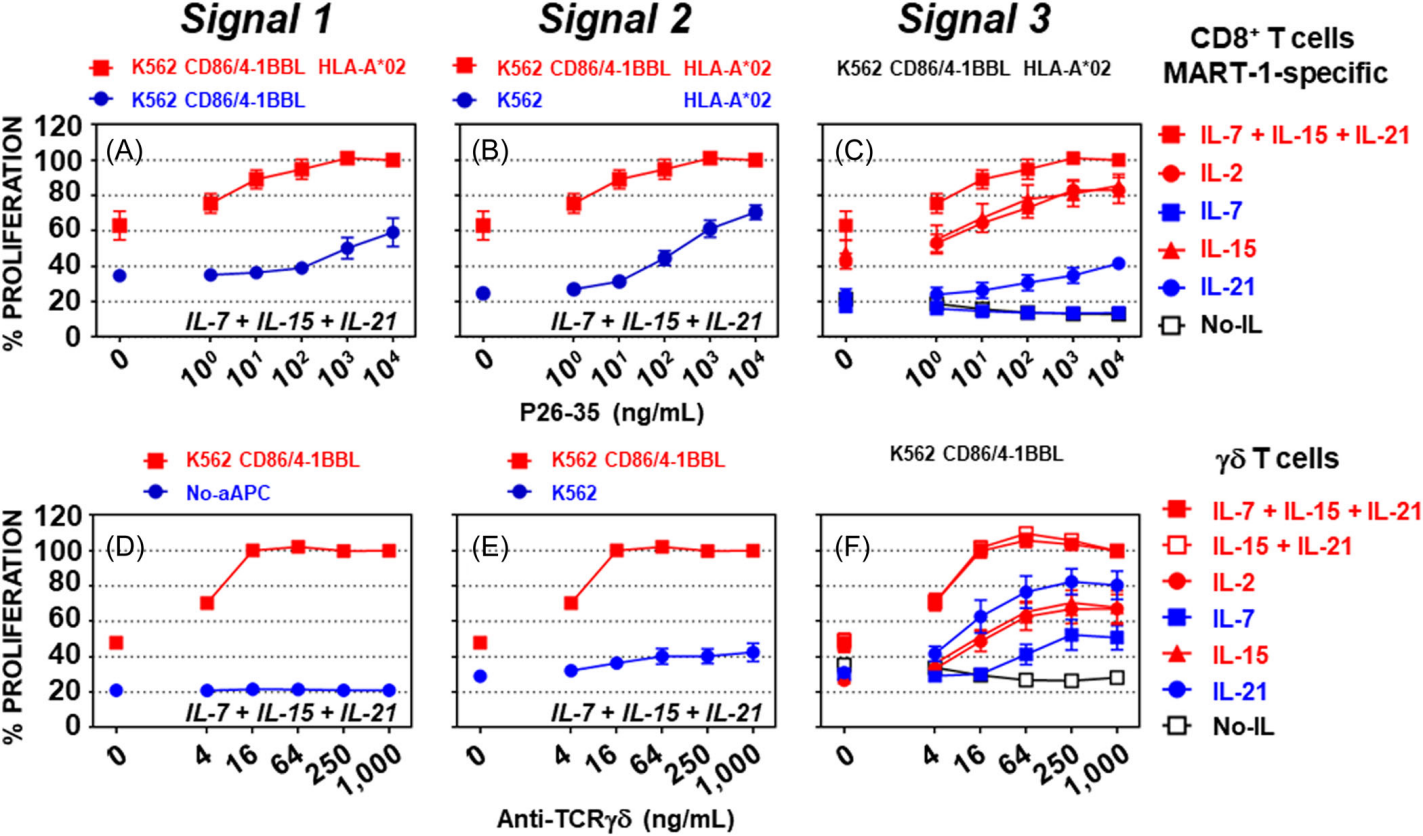
Human CD8^+^ and γδ T cell proliferation and activation relies on three signals. Proliferation of CD8^+^ T cells in the presence of serial dilutions of peptide P26‐35, and (A) either CD86/4‐1BBL/HLA‐A*02‐expressing (red squares) or CD86/4‐1BBL‐expressing (blue circles) K562 cells and cytokines (IL‐7 + IL‐15 + IL‐21) (*n* = 5); (B) either CD86/4‐1BBL/HLA‐A*02‐expressing (red squares) or HLA‐A*02‐expressing (blue circles) K562 cells and cytokines (*n* = 5); and (C) CD86/4‐1BBL/HLA‐A*02‐expressing K562 cells, and the absence (open black squares) or presence of IL‐2 (red circles), IL‐7 (blue squares), IL‐15 (red triangles), IL‐21 (blue circles), or IL‐7 + IL‐15 + IL‐21 (red squares) (*n* = 5). Proliferation of γδ T cells in the presence of serial dilutions of an anti‐human TCR γδ antibody, and (D) either the absence (blue circles) or presence (red squares) of K562‐derived aAPC expressing CD86 and 4‐1BBL and cytokines (*n* = 6); (E) either parental (blue circles) or CD86/4‐1BBL‐expressing (red squares) K562 cells and cytokines (*n* = 6); and (F) K562‐derived aAPC expressing CD86 and 4‐1BBL, and either the absence (open black squares) or presence of IL‐2 (red circles), IL‐7 (blue squares), IL‐15 (red triangles), IL‐21 (blue circles), IL‐15 + IL‐21 (open red squares) or IL‐7 + IL‐15 + IL‐21 (solid red squares) (*n* = 6). Data is shown as the mean ± SEM. Normalization of the data and statistical analysis were performed as described in the Section Methods, 2.

**Figure 2:**
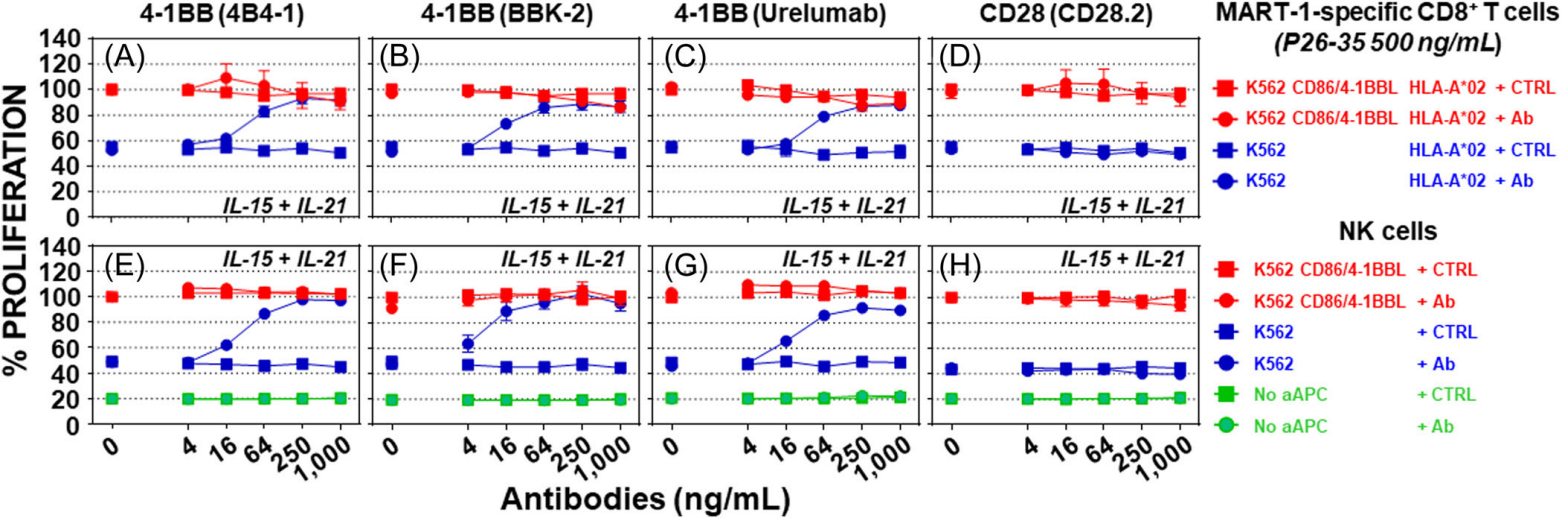
To trigger CD8^+^ T and NK cell proliferation, costimulation by 4‐1BBL is superior to CD28. Proliferation of MART‐1‐specific HLA‐A*02‐restricted CD8^+^ T cells in the presence of peptide P26‐35 (500 ng/ml), cytokines (IL‐15 + IL‐21), and the indicated concentrations of: (A) soluble anti‐4‐1BB antibody clone 4B4‐1 (circles) or mIgG1 isotype controls (squares) (*n* = 5); (B) soluble anti‐4‐1BB antibody clone BBK‐2 (circles) or mIgG1 isotype controls (squares) (*n* = 5); (C) soluble anti‐4‐1BB antibody clone Urelumab (circles) or hIgG4 isotype controls (squares) (*n* = 5); and (D) soluble anti‐CD28 antibody (circles) or mIgG1 isotype control (squares) (*n* = 5). Proliferation of freshly purified NK cells in the presence of cytokines (IL‐15 + IL‐21), the absence (green symbols) or presence of either CD86/4‐1BBL‐expressing (red symbols) or parental (blue symbols) K562, and the indicated concentrations of: (E) soluble anti‐4‐1BB antibody clone 4B4‐1 (circles) or mIgG1 isotype controls (squares) (*n* = 28); (F) soluble anti‐4‐1BB antibody clone BBK‐2 (circles) or mIgG1 isotype controls (squares) (*n* = 11); (G) soluble anti‐4‐1BB antibody clone Urelumab (circles) or hIgG4 isotype controls (squares) (*n* = 15); and (H) soluble anti‐CD28 antibody (circles) or mIgG1 isotype controls (squares) (*n* = 10). Data is shown as the mean ± SEM. Normalization of the data and statistical analysis were performed as described in the Section Methods, 2.

For each experiment, the means of duplicate measurements were calculated and pooled. If one of the compared groups was used at a dose or ratio of 0 for normalization, dose 0 was not included in the analysis. If the normality assumption was verified, a two‐way ANOVA with random effects (and repeated measures, if needed) was performed with heterogeneous variance structures across the groups. In the case of normality assumption violation, a two‐way ANOVA with random effects (and repeated measures, if needed) was performed on the log‐transformed data (Figures 1 and 2) with a heterogeneous variance structure across groups. Depending on the objectives, each analysis was followed by contrast analyses to test the comparisons of interest, followed by Bonferroni‐Holm or Tukey adjustments for multiplicity issues. Statistical data were computed using SAS® version 9.4 for Windows 10. Differences were considered significant when the *p* < 0.05.

## 3 RESULTS

### 3.1 Human CD8^+^ and γδ T cell proliferation and activation relies on three signals

In addition to the two necessary signals previously identified for CD4^+^ T cell activation, evidence in literature suggests that a third signal is required to activate CD8^+^ T cells.^30^, ^31^, ^32^, ^33^ Consequently, K562‐derived aAPCs could be engineered to provide signal 1 (MHC I/peptide complexes) with or without signal 2 (costimulatory ligand CD86 and 4‐1BBL). The requirement for a third signal was evaluated by testing cytokines IL‐7, IL‐15, and IL‐21.^34^ In this investigation, a MART‐1‐specific HLA‐A*02‐restricted CD8^+^ T cell line was established in culture (as described in the Section Methods, 2) and maintained for 22 weeks (Supporting Information: Figure S5A). Most T cells were CD8^+^ and stained positive with the HLA‐A*02/P26‐35 dextramer but negative with the HLA‐A*02/P157‐164‐V dextramer (Supporting Information: Figure S5B). The HLA‐A2 positive K562 target cells were determined to be specifically killed by MART‐1‐specific HLA‐A*02‐restricted CD8^+^ T cells in the presence of peptide P26‐35 (Supporting Information: Figure S5C).

To highlight the role of signal 1, CD8^+^ T cell proliferation was compared in the presence of 50‐Gy x‐irradiated K562‐derived aAPCs expressing either CD86 and 4‐1BBL or CD86, 4‐1BBL, and HLA‐A*02, and serial dilutions of the peptide P26‐35, and cytokines (Figure 1A). As expected, in the presence of cytokines, a strong peptide dose‐dependent proliferation was observed when CD86, 4‐1BBL, and HLA‐A*02 were co‐expressed (Figure 1A, *p* < 0.0001). The requirement for signal 2 was assessed by comparing CD8^+^ T cell proliferation in the presence of 50‐Gy x‐irradiated HLA‐A*02‐positive K562 cells expressing or not expressing CD86 and 4‐1BBL, in the presence of cytokines and serial dilutions of peptide P26‐35 (Figure 1B). Stronger peptide‐dose‐dependent CD8^+^ T cell proliferation was observed when CD86 and 4‐1BBL were expressed, highlighting the importance of costimulation in CD8^+^ T cell expansion (Figure 1B, *p* < 0.001). The low T cell proliferation supported by the HLA‐A2‐positive K562 may, at least partially, be due to the low expression of 4‐1BBL by the parental K562 (Supporting Information: Figure S2), providing a lower level of costimulation (see below). Finally, signal 3 was tested in the presence of the 50‐Gy x‐irradiated K562‐derived aAPCs expressing CD86, 4‐1BBL, and HLA‐A*02, with serial dilutions of peptide P26‐35, and in the presence or absence of cytokines alone or in combination (Figure 1C). In the absence of cytokines or in the presence of IL‐7, no proliferation was detected (Figure 1C, *p* > 0.2). IL‐21 triggered a small but significant amount of T‐cell proliferation (*p* < 0.001 from 10 to 10,000 ng/mL of peptide P26‐35). While IL‐2 (*p* < 0.0001), IL‐15 (*p* < 0.0001), and a combination of IL‐7 + IL‐15 + IL‐21 (*p* < 0.0001) all triggered strong peptide‐dose‐dependent CD8^+^ T cell proliferation, demonstrating that the presence of specific inflammatory cytokines is mandatory for the division of CD8^+^ T cells (Figure 1C). No difference was detected between IL‐2 and IL‐15 (*p* > 0.7), and the IL‐7 + IL‐15 + IL‐21 combination was only slightly better when compared with IL‐2 (*p* < 0.05 at 1, 10, and 10,000 ng/mL of peptide P26‐35) and IL‐15 alone (*p* < 0.05 at 1, 10, and 10,000 ng/mL of peptide P26‐35; Figure 1C). The engagement of the TCRs by the MHC I/peptide complexes (signal 1) and costimulatory signaling (signal 2) was determined to be insufficient for the induction of CD8^+^ T cell proliferation, without a third cytokine induced signal. This showed that all three signals are required to trigger optimal CD8^+^ T‐cell proliferation.

To decipher the signals required to trigger human γδ T cell proliferation and activation, HLA‐A2 negative aAPCs expressing the costimulatory ligands CD86 and 4‐1BBL were used in the presence of the panel of cytokines as previously described, and TCR engagement was mimicked using an antihuman TCR γδ antibody. To shed light on the requirement for signal 1, the proliferation of freshly purified γδ T cells from six different human donors was compared in either the presence or absence of 50‐Gy x‐irradiated K562‐derived aAPCs expressing CD86 and 4‐1BBL, with cytokines and serial dilutions of the anti‐human TCR γδ antibody (Figure 1D). Strong antibody dose‐dependent proliferation was observed only in the presence of K562 expressing CD86 and 4‐1BBL (Figure 1D, *p* < 0.0001). To evaluate the requirement for signal 2, the parental K562 cells were compared to the K562‐derived aAPCs expressing CD86 and 4‐1BBL in the presence of cytokines and serial dilutions of the anti‐human TCR γδ antibody (Figure 1E). Although minimal proliferation was detected in the presence of the parental K562 cell line (blue circles, Figure 1E vs. Figure 1D, *p* < 0.0001 from 16 to 1,000 ng/ml of anti‐TCR γδ antibody), human γδ T cell proliferation was maximal in the presence of K562‐derived aAPCs expressing CD86 and 4‐1BBL (Figure 1E, *p* < 0.0001). The requirement for signal 3 was assessed using 50‐Gy x‐irradiated K562‐derived aAPCs expressing CD86 and 4‐1BBL, with serial dilutions of the anti‐human TCR γδ antibody, and in the absence or presence of cytokines, either alone or in combination (Figure 1F). In the absence of cytokines, no proliferation was detected (Figure 1F). Optimal human γδ T cell proliferation was observed in the presence of either IL‐15 + IL‐21 (*p* < 0.0001) or IL‐7 + IL‐15 + IL‐21 (*p* < 0.0001) with no apparent differences between them (Figure 1F, P∼1.0). At the highest concentrations of the anti‐TCR γδ antibody, IL‐2 (*p* < 0.0001), IL‐7 (*p* < 0.001), IL‐15 (*p* < 0.0001), and IL‐21 (*p* < 0.0001), could all sustain human γδ T cell proliferation (Figure 1F). The results are similar to those obtained for CD8^+^ T cell proliferation (expressing a TCR αβ), as the TCR (signal 1) plus costimulatory signaling (signal 2), were not able to induce γδ T cell proliferation without the third cytokine induced signal. Consequently, it was determined that all three signals are required to trigger optimal γδ T cell proliferation.

### 3.2 To trigger CD8^+^ T and NK cell proliferation, costimulation by 4‐1BBL is superior to CD28

To evaluate the roles of CD28 and 4‐1BB in CD8^+^ T cell costimulation, a T cell proliferation assay was performed by co‐culturing MART‐1‐specific HLA‐A*02‐restricted CD8^+^ T cells with 50‐Gy x‐irradiated HLA‐A*02‐positive K562‐derived aAPCs, in the presence of cytokines, soluble peptide P26‐35, and one of the three independent clones of anti‐4‐1BB (4B4‐1, BBK‐2, or urelumab) or a clone of the anti‐CD28 (CD28.2) antibody, to mimic the activity of either membrane‐bound 4‐1BBL or CD86 expressed on aAPCs (Figure 2). K562‐derived aAPCs expressing CD86, 4‐1BBL, and HLA‐A*02 were used as positive controls. The 4B4‐1 (Figure 2A, *p* = 1.0), BBK‐2 (Figure 2B, *p* = 1.0), and urelumab (Figure 2C, *p* > 0.05 at all concentrations except for 250 ng/ml, *p* < 0.05) clones did not affect the expansion of MART‐1‐specific CD8^+^ T cells in the presence of HLA‐A*02‐positive K562 cells forced to express CD86 and 4‐1BBL molecules. However, the 4B4‐1 (Figure 2A, *p* < 0.0001), BBK‐2 (Figure 2B, *p* < 0.0001) and urelumab (Figure 2C, *p* < 0.001) clones, increased the MART‐1‐specific CD8^+^ T cell proliferation in a dose‐dependent manner, when HLA‐A*02‐positive K562 cells were used as aAPCs. The anti‐CD28 antibody had no effect on MART‐1‐specific CD8^+^ T cell proliferation, regardless of the CD86 and 4‐1BBL expression by the aAPCs (Figure 2D, *p* = 1.0). Therefore, it was concluded that the engagement of 4‐1BB is mandatory to support MART‐1‐specific HLA‐A*02‐restricted CD8^+^ T cell proliferation in the presence of inflammatory cytokines.

Similarly, for NK cell proliferation, CD28 and 4‐1BB costimulations were assessed in a NK cell proliferation assay where freshly purified NK cells were cocultured in the absence or presence of the 50‐Gy x‐irradiated parental K562 cell line or K562‐derived aAPCs expressing CD86 and 4‐1BBL, in the presence of cytokines, and either one of the three independent clones of anti‐4‐1BB (4B4‐1, BBK‐2, or urelumab) or one clone of the anti‐CD28 (CD28.2) antibodies, to mimic the activity of the membrane‐bound 4‐1BBL or CD86 expressed on the aAPCs, respectively. K562‐derived aAPCs expressing CD86 and 4‐1BBL were used as positive controls. The 4B4‐1 (Figure 2E, *p* = 1), BBK‐2 (Figure 2F, *p* = 1.0), and urelumab (Figure 2G, *p* > 0.05) clones did not affect the expansion of the NK cells in the presence of K562‐derived aAPCs forced to express CD86 and 4‐1BBL molecules. However, the 4B4‐1 (Figure 2E, *p* < 0.0001), BBK‐2 (Figure 2F, *p* < 0.0001), and urelumab (Figure 2G, *p* < 0.001) clones, increased NK cell proliferation in a dose‐dependent manner, when the parental K562 cells were used as an aAPC. The anti‐CD28 antibody had no effect on NK cell proliferation regardless of the CD86 and 4‐1BBL expression by the aAPCs (Figure 2H, *p* > 0.05). In the absence of aAPCs (Figures 2E,F,G,H, *p* > 0.05), NK cell proliferation was undetectable regardless of the presence of anti‐4‐1BB and anti‐CD28 antibodies. Therefore, 4‐1BB engagement is essential to support NK cell proliferation in the presence of parental K562 cells and inflammatory cytokines.

### 3.3 Effector killer cells are insensitive to cytokine absence and the engagement of 4‐1BB and CD28

**Figure 3:**
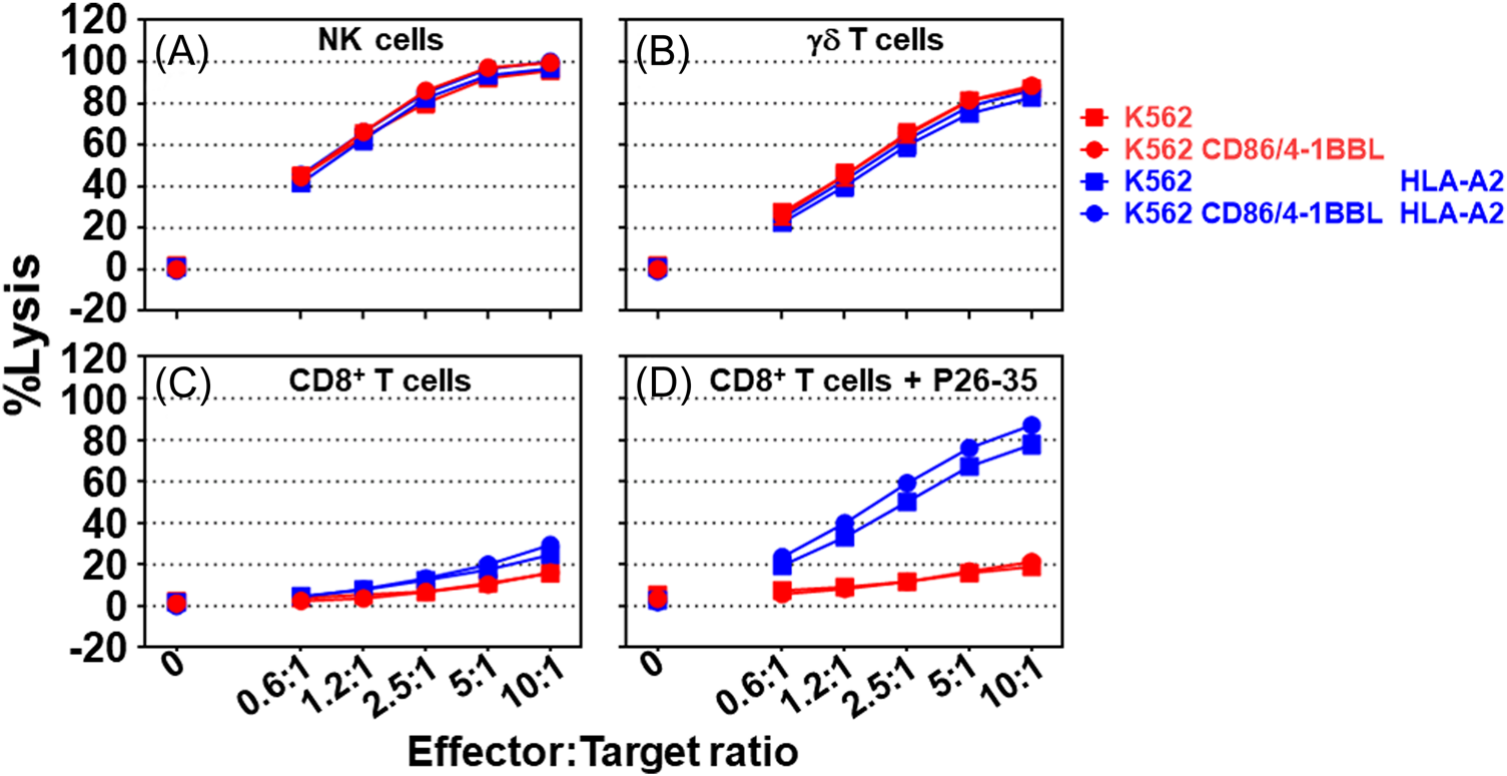
Effector killer cells are insensitive to cytokine absence and the engagement of 4‐1BB and CD28. The cytolytic activity of effector (A) NK, (B) γδ T, and MART‐1‐specific HLA‐A*02‐restricted CD8^+^ T cells in both the (C) absence and (D) presence of peptide P26‐35 (500 ng/mL) against the parental K562 (red squares), CD86/4‐1BBL‐expressing K562 (red circles), HLA‐A*02‐expressing K562 (blue squares), or CD86/4‐1BBL/HLA‐A*02‐expressing K562 (blue circles) target cells in a 4‐h calcein release assay at different effector to target cell ratios (E:T) in 96‐well flat‐bottom plates in duplicate. Data is shown as the mean ± SEM (*n* = 5). Normalization of the data and statistical analysis were performed as described in the Section Methods, 2.

To evaluate the role of costimulation on the sensitivity of the target cells to effector killer cells, parental or HLA‐A*02‐positive K562 cells were compared to target cells forced to express CD86 and 4‐1BBL. Effector killer cells were generated in 1 week (as described in the Section Methods, 2) and were extensively washed to eliminate the cytokines before co‐culturing with the target cells. NK (Figure 3A, *p* > 0.05 at all ratios and regardless of the comparison performed) and γδ T cells (Figure 3B, *p* > 0.05 at all ratios and regardless of the comparison performed) killed the four target cells irrespective of HLA‐A*02, CD86, and 4‐1BBL expression. Furthermore, K562 target cells were found to be insensitive to the MART‐1‐specific HLA‐A*02‐restricted CD8^+^ T cell cytolytic activity in the absence of the P26‐35 peptide regardless of HLA‐A*02, CD86, and 4‐1BBL expression (Figure 3C). In the absence of HLA‐A*02 expression, K562 target cells were insensitive to MART‐1‐specific CD8^+^ T cell cytolytic activity, even though the P26‐35 peptide was present (Figure 3D). When HLA‐A*02 was expressed by the target cells (Figure 3D), target cell lysis by MART‐1‐specific HLA‐A*02‐restricted CD8^+^ T cells relied on the presence of P26‐35 (Figure 3D, *p* < 0.0001) regardless of the CD86 and 4‐1BBL expression. The sensitivity of the target cells to the cytolytic activity of the effector NK, γδ T, and CD8^+^ T cells was determined to be independent of costimulation (signal 2) and the cytokines (signal 3).

### 3.4 Sensitivity of melanoma and multiple myeloma tumor cells to cytolytic effector killer cells activity

**Figure 4:**
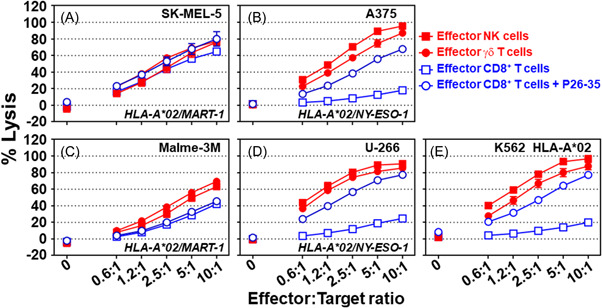
Sensitivity of melanoma and multiple myeloma tumor cells to cytolytic effector killer cell activity. The cytolytic activity of effector NK (solid red squares), γδ T (solid red circles), and MART‐1‐specific HLA‐A*02‐restricted CD8^+^ T cells in either the presence (open blue circles) or absence (open blue squares) of the peptide P26‐35 (500 ng/ml) against the tumor cell lines (A) SK‐MEL‐5, (B) A375, (C) Malme‐3M, and (D) U‐266, and the (E) HLA‐A*02‐expressing K562 target cells in a 4‐h calcein release assay at different E:T ratios in 96‐well flat‐bottom plates in duplicate. Data is shown as the mean ± SEM (*n* = 4). Normalization of the data and statistical analysis were performed as described in the Section Methods, 2.

SK‐MEL‐5, Malme‐3M, A375, and U‐266 tumor cell lines were all previously shown to display HLA‐A*02 and express MART‐1 protein (SK‐MEL‐5 and Malme‐3M), and was confirmed using flow cytometry and western blot analysis (Supporting Information: Figure S3). Effector killer cells were prepared, and the calcein‐release assay was performed, as described above. SK‐MEL‐5 (Figure 4A), A375 (Figure 4B), Malme‐3M (Figure 4C), U‐266 (Figure 4D), and K562 HLA‐A*02 (Figure 4E) were sensitive to the cytolytic activity of the effector NK and γδ T cells to varying degrees. MART‐1 protein‐expressing SK‐MEL‐5 (Figure 4A, *p* > 0.05) and Malme‐3M (Figure 4C, *p* > 0.05) tumor cells were sensitive to MART‐1‐specific CD8^+^ T cell cytolytic activity, regardless of the presence of the P26‐35 peptide. MART‐1 negative A375 (Figure 4B, *p* < 0.0001), U‐266 (Figure 4D, *p* < 0.0001), and HLA‐A*02‐positive K562 (Figure 4E, *p* < 0.001) cells were all resistant to MART‐1‐specific CD8^+^ T cell cytolytic activity, unless the soluble P26‐35 peptide was added. Except for SK‐MEL‐5 target cells, where no gross differences were observed between NK, γδ T, and MART‐1‐specific HLA‐A*02‐restricted CD8^+^ T cell cytolytic activity, the NK and γδ T cells were slightly, but significantly, more cytotoxic than MART‐1‐specific HLA‐A*02‐restricted CD8^+^ T cells. These results show that MART‐1 protein‐expressing tumor SK‐MEL‐5 and Malme‐3M generate MHC I/P26‐35 complexes, which are recognized by MART‐1‐specific HLA‐A*02‐restricted CD8^+^ T cells. Although the cytolytic activity of the MART‐1‐specific HLA‐A*02‐restricted CD8^+^ T cell line relies on the expression of HLA‐A*02 and MART‐1, all the tumors tested were sensitive to killing by the effector NK, γδ T, and MART‐1‐specific HLA‐A*02‐restricted CD8^+^ T cells, to varying degrees.

### 3.5 Cytolytic activity of resting versus effector NK cells

**Figure 5:**
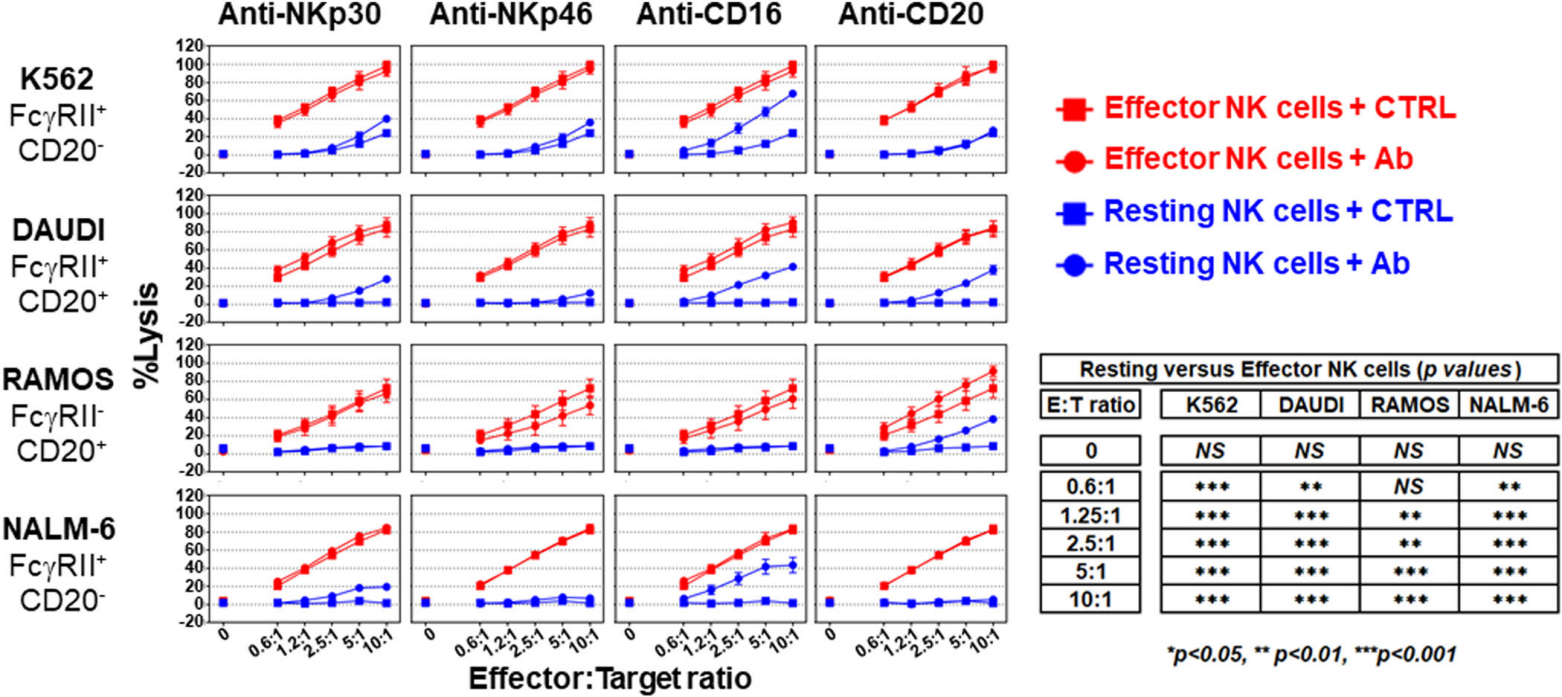
Cytolytic activity of resting versus effector NK cells. The cytolytic activity of effector (red symbols) and resting (blue symbols) NK cells in the presence of 5 µg/ml of either the indicated antibody (circles) or its isotypic control (squares) against the K562, DAUDI, RAMOS, or NALM‐6 target cells in a 4‐h calcein release assay at different E:T ratios in 96‐well flat‐bottom plates in duplicate. Data is shown as the mean ± SEM (*n* = 4). Normalization of the data and statistical analysis were performed as described in the Section Methods, 2.

NK cells, particularly CD56^dim^CD16^+^, can kill stressed, diseased (natural cytotoxicity), or antibody‐coated cells via a phenomenon known as antibody‐dependent cellular cytotoxicity (ADCC). Fc receptor‐positive (FcγRII, CD32) target cells can also be lysed by NK cells in the presence of an antibody directed against an NK cell‐activating receptor (redirected cell lysis or reverse ADCC).^35^ A set of human target cells expressing CD32 (K562, DAUDI, and NALM‐6) and/or CD20 (DAUDI and RAMOS) (Supporting Information: Figure S6) were tested for their sensitivity to killing by resting or effector NK cells, either in the presence or absence of antibodies known to trigger ADCC (anti‐CD20) or reverse ADCC (anti‐NKp30, anti‐NKp46, and anti‐CD16) (Figure 5). Effector NK cells exhibited stronger cytolytic activity than resting NK cells, regardless of the target tested (Figure 5). In the absence of antibodies, all the tumor cell lines were resistant to the cytolytic activity of the resting NK cells, except for the K562 cell line, which was slightly sensitive. Activation of CD16 by either ADCC (anti‐CD20 antibody) or reverse ADCC (anti‐CD16 antibody) triggered NK cell cytolytic activity. Engagement of NKp30 or NKp46 by their respective antibodies, marginally increased the killing of K562 (*p* < 0.001 at ratios of 5:1 and 10:1 for NKp30 and NKp46), DAUDI (*p* < 0.001 at a ratio of 10:1 for NKp30 and NKp46, and *p* < 0.001 at a ratio of 5:1 for NKp30), and NALM‐6 (*p* < 0.001 at ratios of 5:1 and 10:1 for NKp30) by the resting NK cells. Effector NK cells in the presence of control antibodies were more potent in killing target tumor cells (K562, DAUDI, and NALM‐6) than resting NK cells, regardless of the NKp30, NKp46, or CD16 engagement. As expected, the RAMOS tumor cell line lacking CD32 expression was insensitive to redirected cell lysis mediated by anti‐CD16, ‐NKp30, or ‐NKp46 antibodies. These data show that CD16 is the most potent receptor capable of activating the cytolytic activity of resting NK cells, and suggests that NK cells exist in two states, resting versus effector, and the latter has a greater capacity to kill tumor cells.

### 3.6 By default, resting NK, γδ T, and CD8^+^ T cells are devoid of cytolytic activity

**Figure 6:**
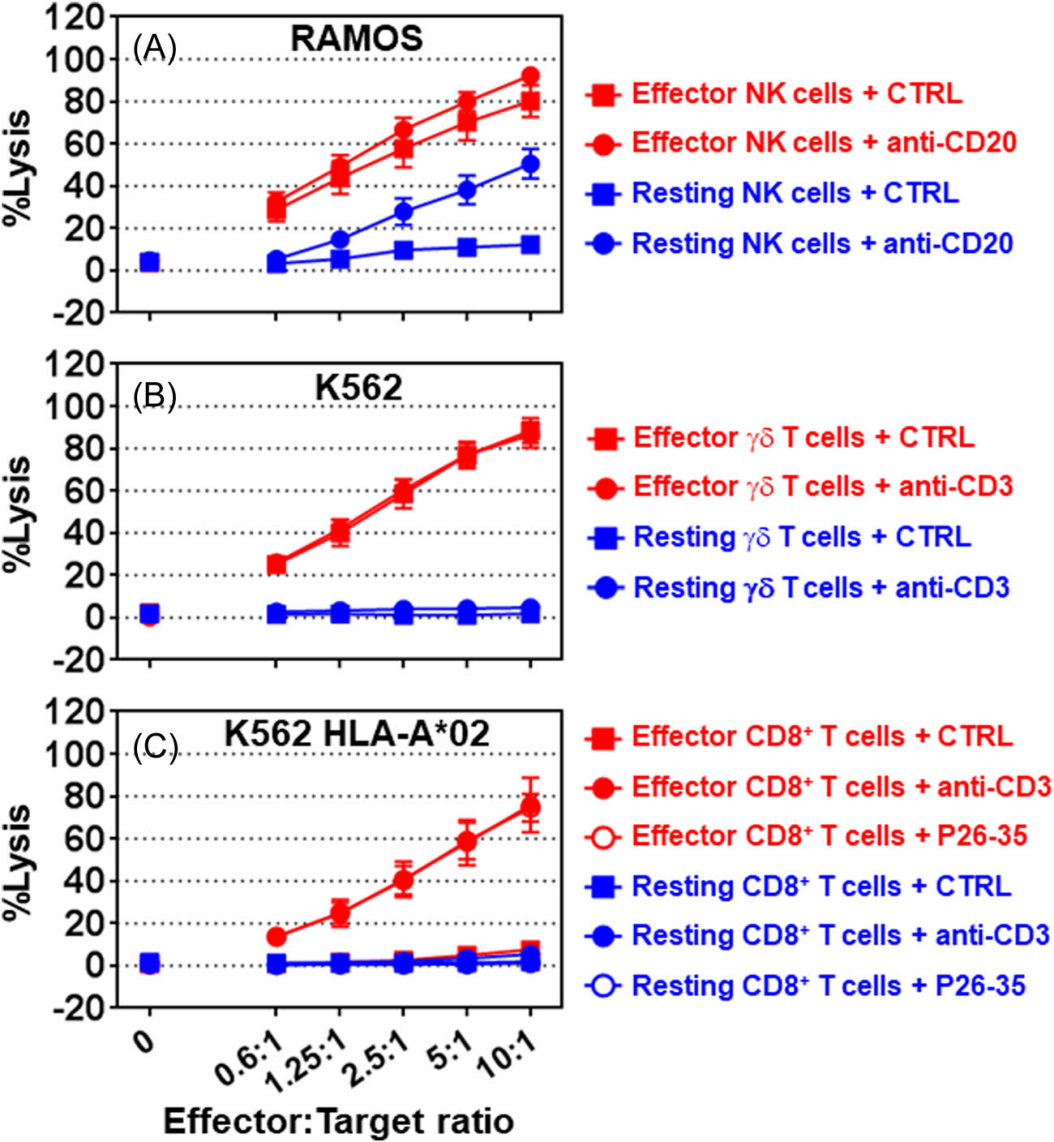
By default, resting NK, γδ T, and CD8^+^ T cells are devoid of cytolytic activity. The cytolytic activity of the effector (red symbols) and resting (blue symbols) (A) NK, (B) γδ T, and (C) CD8^+^ T cells in either the presence of 5 µg/ml of the indicated antibody (solid circles) or its isotypic control (solid squares), or 500 ng/ml of peptide P26‐35 (open circles) against RAMOS (*n* = 7) (A), K562 (*n* = 5) (B), or HLA‐A*02‐expressing K562 (*n* = 4) (C) target cells in a 4‐h calcein release assay at different E:T ratios in 96‐well flat‐bottom plates in duplicate. Data is shown as the mean ± SEM. Normalization of the data and statistical analysis were performed as described in the Section Methods, 2.

Most tumor cell lines are resistant to the cytolytic activity of resting NK cells (Figure 5) but are highly sensitive to their effector counterparts. To determine whether this difference was also applied to the other lymphocyte killer cells evaluated in this study, the cytolytic activities of freshly purified (resting) NK (Figure 6A), γδ T (Figure 6B), and CD8^+^ T (Figure 6C) cells were compared to those that were expanded in vitro in the presence of the three mandatory signals. The RAMOS tumor cell line was insensitive to resting NK cell cytolytic activity unless coated with an anti‐CD20 antibody (Figure 6A, *p* < 0.0001 at ratios of 2.5:1 to 10:1). Effector NK cells were more effective than resting NK cells at killing RAMOS tumor cells (Figure 6A, *p* < 0.0001 at all ratios), regardless of whether the anti‐CD20 antibody was present, and resting γδ T cells were unable to kill K562 tumor cells, regardless of the presence of an anti‐CD3 antibody (Figure 6B, *p* > 0.3 at all ratios). Effector γδ T cells were robust killers of K562 tumor cells (Figure 6B, *p* < 0.0001 at ratios of 1.2:1 to 10:1) regardless of the presence of an anti‐CD3 antibody. Similarly, resting CD8^+^ T cells were unable to kill HLA‐A*02‐positive K562 tumor cells, regardless of the presence of an anti‐CD3 antibody (Figure 6C, *p* > 0.05). However, MART‐1‐specific HLA‐A*02‐restricted CD8^+^ T cells were effective killer cells in the presence of the P26‐35 peptide (Figure 6C, *p* < 0.0001 at ratios of 1.2:1 to 10:1) or an anti‐CD3 antibody (Figure 6C, *p* < 0.0001 at ratios of 1.2:1 to 10:1). Activation of MART‐1‐specific HLA‐A*02‐restricted CD8^+^ T cell cytolytic activity either with peptide P26‐35 or the engagement of CD3 by the OKT3 antibody was indistinguishable (Figure 6C, *p* = 1.0). These results indicate that the resting NK, γδ T, and CD8^+^ T cells are devoid of effector functions and may be considered precursors of the effector killer cells. For effector CD8^+^ T cells, TCR engagement is mandatory to kill target cells specifically and selectively. Effector NK and γδ T cells are formidable killers, but the receptor(s) involved in target recognition are currently unknown.

## 4 DISCUSSION

To break tolerance, which is a default hallmark of the steady state, three signals including one for danger^36^ are required as follows: (i) close cell‐to‐cell contact, involving interactions between MHC/peptide complexes and TCR for CD8^+^ T cells, which is still poorly defined at the molecular level for NK and γδ T cells; (ii) costimulation through the engagement of 4‐1BB by its ligand 4‐1BBL; and (iii) cytokine receptor signaling induced by IL‐15 and IL‐21. These three signals trigger the proliferation and differentiation of resting NK, γδ T, and CD8^+^ T cells into effector cells, to ensure that they reach the critical number required to fight and eliminate dangerous pathogens, their cellular reservoirs, and tumor cells. Once differentiated into effector cells, these lymphocytes can kill pathogens, infected and/or tumor cells, regardless of the costimulation and inflammatory cytokines, and according to Fuchs and Matzinger in 1996,^37^ “no tissue of the body is immune from T cell‐mediated cytolysis.”

The human body cannot host an adequate number of different T cells to cover the entire set of MHC/peptide complexes.^38^ Therefore, to achieve TCR repertoire completeness, a single TCR must be able to recognize many different MHC/peptide complexes.^39^ This requisite promiscuity of TCR has compelled the immune system to evolve to protect the host from fatal infection while avoiding lethal autoimmunity.^37^ For example, cortical DP thymocytes cannot undergo further development in the absence of TCR engagement,^40^, ^41^ and T cell survival at the periphery is supported by TCR promiscuity.^42^, ^43^, ^44^ To avoid autoimmunity, CD8^+^ T cells that exit the thymus must be inactive (Figure 6C) and tightly regulated to prevent host injury, as predicted by the danger paradigm.^36^, ^37^ The three‐signal model illustrated in Figure 1, and the requirement of a third signal was initially proposed by Curtsinger et al.^30^ in 1999 and gained wider support,^32^, ^33^ as a trigger for the proliferation and activation of CD8^+^ T cells.

It appears likely that the danger signal brings a third signal to the T cells by the secretion of cytokines from specialized innate immune cells, and an unknown mechanism by which the level of engagement of costimulatory receptors such as 4‐1BB on CD8^+^ T cells fine‐tunes IL‐15‐ (± IL‐21‐) driven CD8^+^ T cell proliferation and activation (Figure 2A,B,C). By default, CD8^+^ T cells are tolerant; that is, they are unable to kill a target cell upon TCR engagement (Figure 6C) and their proliferation and activation are not triggered by the co‐engagement of the TCR by the appropriate MHC I/peptide complexes and 4‐1BB costimulatory receptor by its ligand 4‐1BBL, in the absence of IL‐15 (Figure 1C). CD8^+^ T cell proliferation was finely tuned and proportional to the level of 4‐1BB engagement upon TCR and cytokine receptor signaling (Figures 1B and 2A,B,C). Two signals were initially described as triggers of CD4^+^ T‐cell proliferation and activation.^45^ These data and the proposed model, based on the best available knowledge at the time, may have been misinterpreted and do not reflect the shift from tolerance to immunity as proliferation in this setting is autocrine IL‐2‐dependent, although in vivo IL‐2 reduces T cell proliferation and activation^46^ by promoting and supporting Treg functions.^47^ Consistently, IL‐2 production and T cell proliferation were observed before the induction of specific T cell tolerance,^48^ and tolerance, rather than immunity, crucially depends on IL‐2.^49^ In vitro IL‐2 and IL‐15 activities are indissociable when purified CD8^+^ T cells are used (Figure 1C); however, IL‐2 deficiency is associated with lymphoproliferative disorders and autoimmunity.^46^ In contrast, the number of memory CD8^+^ T cells is drastically reduced in IL‐15‐deficient mice.^50^, ^51^ Therefore, this two‐signal model of CD4^+^ T cell proliferation and activation is not representative of the switch from tolerance to immunity, which was initially presumed, as innate immune cells, most likely DCs, must first recognize a danger signal and then send an SOS signal to provide all the necessary signals for initiating T cell proliferation and activation. This was elegantly demonstrated in vivo by Curtsinger et al.^31^ where, in the absence of an adjuvant or IL‐12, a high dose of antigen could trigger CD8^+^ T cell proliferation without effector cytolytic function. Similar proliferation was triggered by a small amount of antigen in the presence of either an adjuvant or IL‐12, concomitantly with the development of the effector cytolytic function. Innate immune cells, through the recognition of a danger signal control adaptive immune cells, and by default, antigen presentation that is associated with or without costimulatory receptor engagement will ultimately lead to tolerance in the absence of an SOS signal (Figure 1C). It is therefore unlikely that peripheral tolerance is maintained by a mechanism referred to as anergy,^11^, ^14^ as cytokines (the third signal described in this manuscript) can rescue T cells from anergy.^14^, ^52^ Consequently, in the presence of three signals, naïve and anergic T cells will ultimately be activated to the same extent. In the absence of signal 3, naïve and anergic T cells remain silent (tolerant)^30^, ^31^, ^32^, ^33^ as predicted by Fuch and Matzinger^37^ in 1996.

Freshly isolated (resting) NK cells have low or null cytolytic activity^22^, ^53^; however, upon recognition of the cell‐bound antibodies (with an effector Fc), CD16 engagement stimulates their killer activity, a phenomenon called ADCC.^53^ ADCC may be seen as a bridge between innate and adaptive immunity, and it is therefore tempting to see some analogies between ADCC and T cell‐mediated cytotoxicity. However, in contrast to TCR engagement, which has no effect on resting CD8^+^ T cell cytolytic activity (Figure 6C), CD16 engagement triggers ADCC mediated by resting NK cells (Figure 6A). Conversely, CD16 engagement had no effect on the cytolytic activity of the effector NK cells (Figure 6A), and TCR engagement triggered killing of the target cells by effector CD8^+^ T cells (Figure 6C). NK cells are more efficiently armed to kill stressed, infected, and tumor cells by cytokines such as IL‐2 and IL‐15 (Figures 5 and 6).^22^ Cytokine starvation of effector NK cells led to a reversal of the natural cytotoxicity back to normal levels while restoring ADCC.^26^

In addition to CD16, the cytolytic activity of NK cells is tightly regulated by a balance between activating and inhibiting receptors.^54^, ^55^ The low or null cytolytic activity of resting NK cells is therefore, a consequence of excess negative versus positive signaling or low or absent activating signals. The engagement of activating receptors such as NKG2D or 2B4 or natural cytotoxicity receptors (NCRs) such as NKp30, NKp44, or NKp46 alone cannot trigger NK cell cytotoxicity unless coupled with another activating receptor and magnified by cytokine signaling,^53^, ^56^ thus strengthening the tight regulation of NK cell cytolytic activity to avoid a detrimental attack on normal body cells. Consistently, the activation provided by the engagement of NKp30 or NKp46 is relatively low or null when compared to CD16 engagement in resting NK cells (Figure 5). When three signals were provided to trigger NK cell proliferation and activation, effector NK cell cytolytic activity outperformed the trigger by the engagement of CD16, NKp30, and NKp46 on resting NK cells (Figure 5). Furthermore, the engagement of CD16,^26^ NKp30, or NKp46 on the effector NK cells had no effect (Figure 5). These data suggest that NK cells, like CD8^+^ T cells, exist in two states: a resting state, where they are unable to kill any cell of the body regardless of infection and transformation, and an effector state, where they are licensed to kill pathogens, their cellular reservoirs, or transformed cells while being benevolent toward normal cells.

*Escherichia coli* infections can stimulate the expression of 4‐1BBL and membrane‐bound IL‐15 by DCs,^57^ enabling these APCs to trigger and support NK cell proliferation^21^ and parodied by the use of 4‐1BBL‐ and IL‐15‐expressing aAPCs to numerically expand NK cells for adoptive cell therapy.^19^, ^26^ Although clonal expansion is a hallmark of adaptive immunity, this process may also be required to expand specific sub‐populations of NK cells. This was illustrated by observations made in C57BL/6 mice, where the preferential expansion of an NK cell subset expressing Ly49H upon MCMV infection but not upon vaccinia infection, resembled the clonal expansion of antigen‐specific T cells.^58^, ^59^ All other things being equal, numerous subsets of NK cells have been described,^60^, ^61^, ^62^ justifying the need for clonal expansion and activation to provide optimal protection against pathogens or cancer; that is, the number of effector NK cells will constitute the strength of the immune response.

γδ T cells are scarce in human peripheral blood lymphocytes (∼4%), but constitute a major T cell subset of intraepithelial lymphocytes (IELs). The antigens that drive their proliferation and activation are largely unknown.^63^ In human and nonhuman primates, the clonal expansion of γδ T cells is triggered by bacterial^64^, ^65^ and viral^66^, ^67^ infections. Similar to αβ T cells, primary and secondary responses were observed in vivo during *Mycobacterium tuberculosis* ^64^ and CMV infection.^67^ According to my observations (Figure 1D), cell‐to‐cell contact with accessory cells is required^23^ to trigger γδ T cell proliferation and activation in a non‐MHC‐restricted manner.^24^, ^68^ The proliferation and activation of γδ T cells in vitro, rely on the presence of IL‐2, IL‐15, and IL‐21,^64^, ^68^ which is in concurrence with the findings of this study using K562‐derived aAPCS expressing CD86 and 4‐1BBL (Figure 1F).

Contradictory findings involving CD28, 4‐1BB, CD27, and/or CD83 costimulation in human and mouse γδ T cell proliferation and activation have been reported.^16^, ^69^, ^70^, ^71^, ^72^, ^73^ This apparently heterogeneous or contradictory dependency on costimulation in γδ T cell proliferation and activation may rely on the diversity and plasticity of γδ T cells,^74^ along with the combination of costimulatory receptors and cytokines that were used in different investigations. Consequently, this point requires further investigation. Overall, the three‐signal model proposed in this manuscript to trigger the proliferation and activation of γδ T cells is congruent with literature, in the absence of one of these signals, the proliferation and activation of γδ T cells is either low or null (Figures 1D,E,F).

IL‐7, IL‐15, and IL‐21 belong to the family of cytokines that share the γ‐chain (CD132) among their receptor subunits. The binding of IL‐15 and IL‐21 induces receptor oligomerization that leads to the activation of the constitutively bound JAK1 and JAK3 kinases. This signaling complex induces the activation of the PI3K, MAPK, and STAT downstream signaling pathways.^75^, ^76^ The activation of JAK kinases preferentially induces the recruitment and activation of either STAT5 or STAT3 to the IL‐2/IL‐15 or IL‐21 receptor, respectively. Expression of 4‐1BB on NK,^77^, ^78^ γδ T,^79^ and CD8^+^ T ^80^ cells is induced by the presence of cytokines and its engagement with 4‐1BB ligand strengthens the activation of the MAPK signaling pathway, activates the NF‐kB pathway, and inhibits apoptosis^81^ by increasing the expression of Bcl‐xL and Bfl‐1.^82^ Besides the activation of the STAT family transcription factors,^83^ cytokines also induce the expression of eomesodermin (EOMES)^84^ and T‐bet,^83^, ^85^ which are known to regulate granzyme and perforin gene expression.^85^, ^86^, ^87^

## 5 CONCLUSION

The data presented in this manuscript shows that at a steady state, NK, γδ T, and CD8^+^ T cells (either naïve or memory) are devoid of effector functions, and can therefore be considered as precursors of the effector NK, γδ T, and CD8^+^ T cells. Three signals are required to trigger their proliferation and activation/differentiation into effector cells. A cell‐to‐cell contact (signal 1), 4‐1BB engagement (signal 2), and the activation of the common γ‐chain cytokine receptor signaling (signal 3) provide a tight regulation to shift tolerance to immunity. Overall, this data challenges the paradigm of anergy and supports the danger signal theory originally proposed by Polly Matzinger, which states that killer cells are tolerant by default, protecting the mammalian body from autoimmunity.

## AUTHOR CONTRIBUTIONS

Laurent Vidard designed the research, performed the experiments, analyzed the data, and wrote the manuscript.

## CONFLICT OF INTEREST

Laurent Vidard is a full‐time employee of Sanofi and holds shares in the company.

## Supporting information

Supporting information.
