# Supplementary material for: 4‐1BB and cytokines trigger human NK, γδ T, and CD8+ T cell proliferation and activation, but are not required for their effector functions
Source: Immun Inflamm Dis. 2022 Dec 15;11(1):e749. doi: 10.1002/iid3.749 (PMC9753824; doi:10.1002/iid3.749)
Supplement: Supplementary file 1 — Supporting information. [file IID3-11-e749-s001.pdf]

Supplementary data

4-1BB and cytokines trigger natural killer, $\gamma\delta$ T, and CD8^{+} T cell proliferation and activation, but are not required for their effector functions

Laurent Vidard

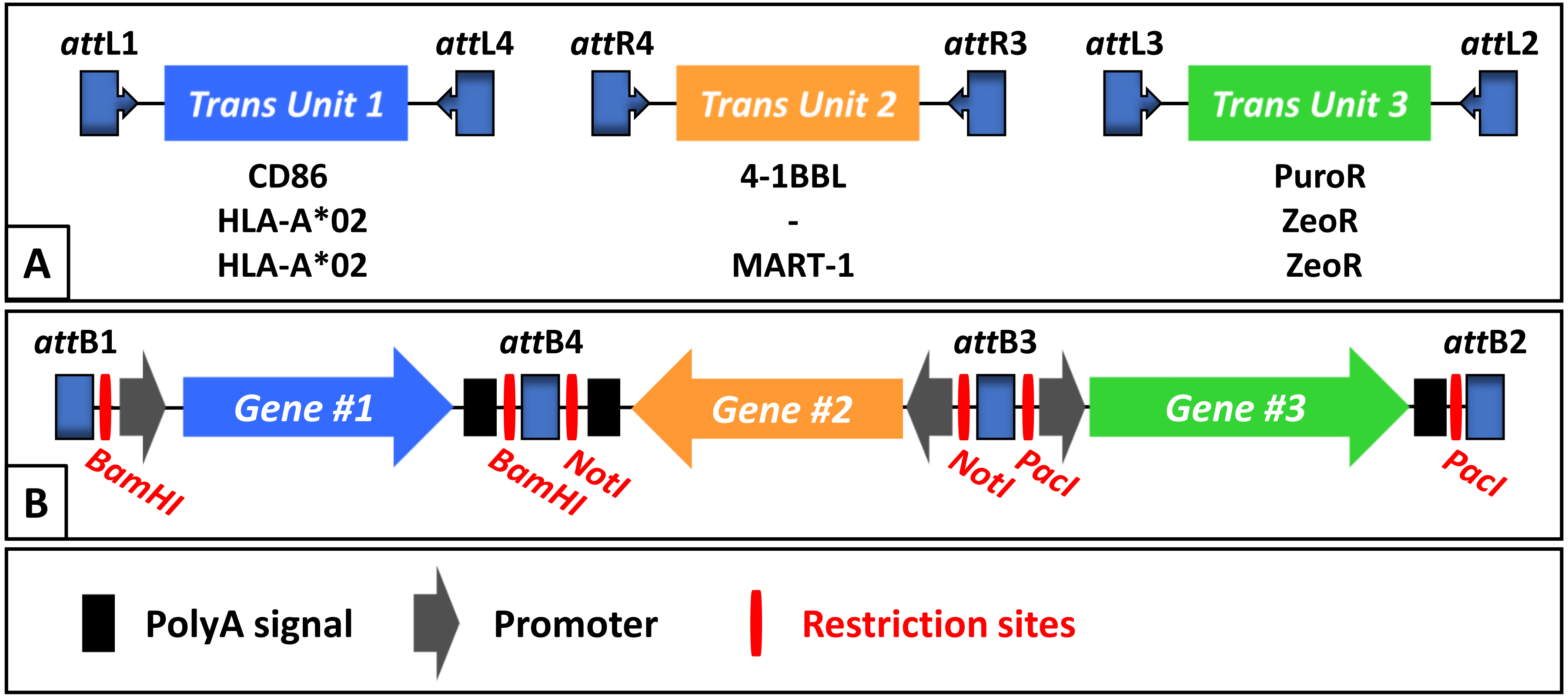

Figure S1: **Schematic representation of the 3-fragment PiggyBac transposon expression vector used to engineer K562-derived aAPCs.** Synthetic genes before (A) and after (B) LR recombination.

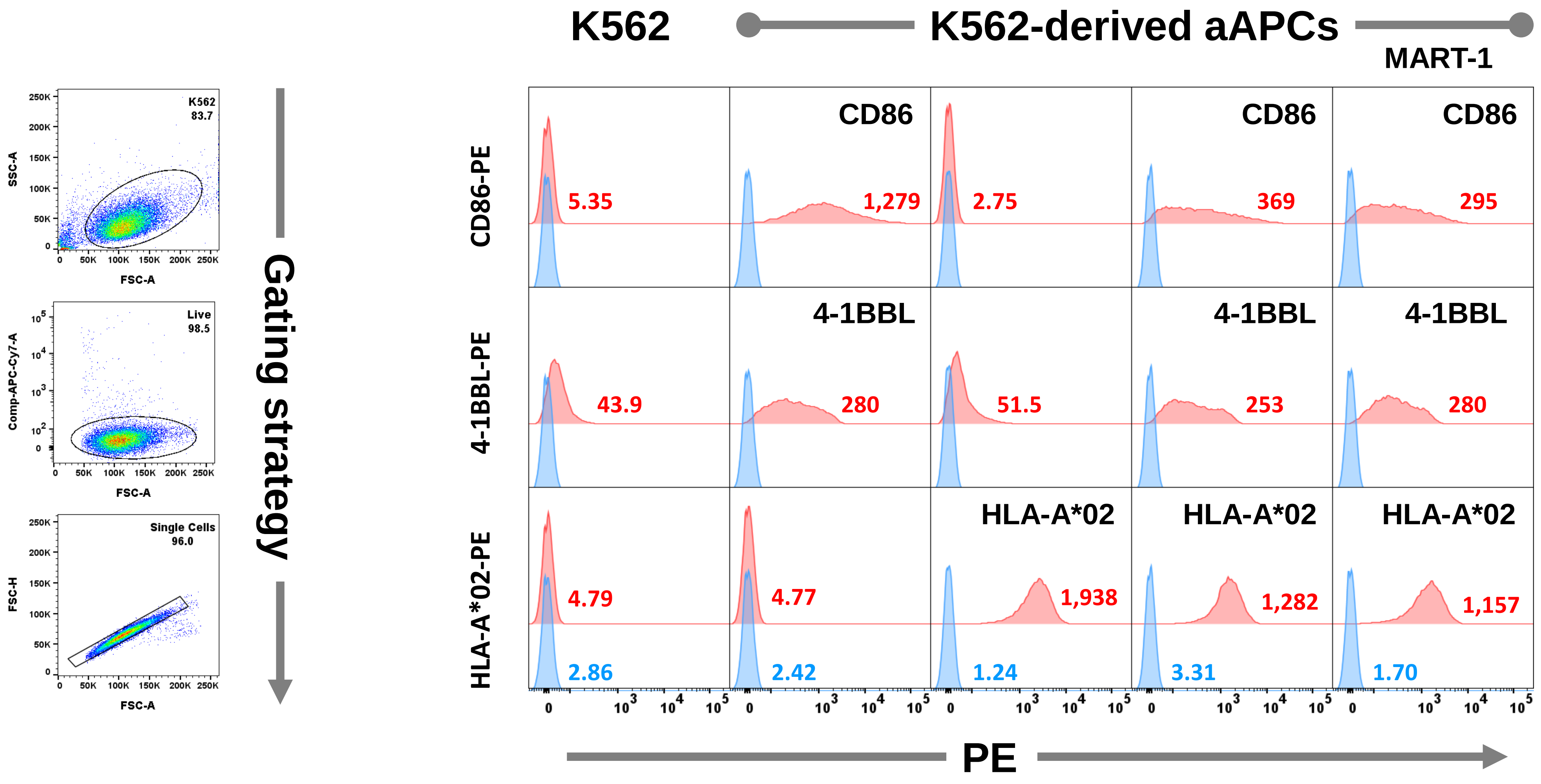

<u>Figure S2</u>: Phenotype of K562-derived aAPCs. Left panel, gating strategy. Right panel, expression of CD86, 4-1BBL and HLA-A*02 by the parental K562 cell line and the different K562-derived aAPCs used in this study. Blue and red numbers indicate the gMFI values of control and tested antibodies, respectively. Data are representative of at least three independent flow cytometry experiments.

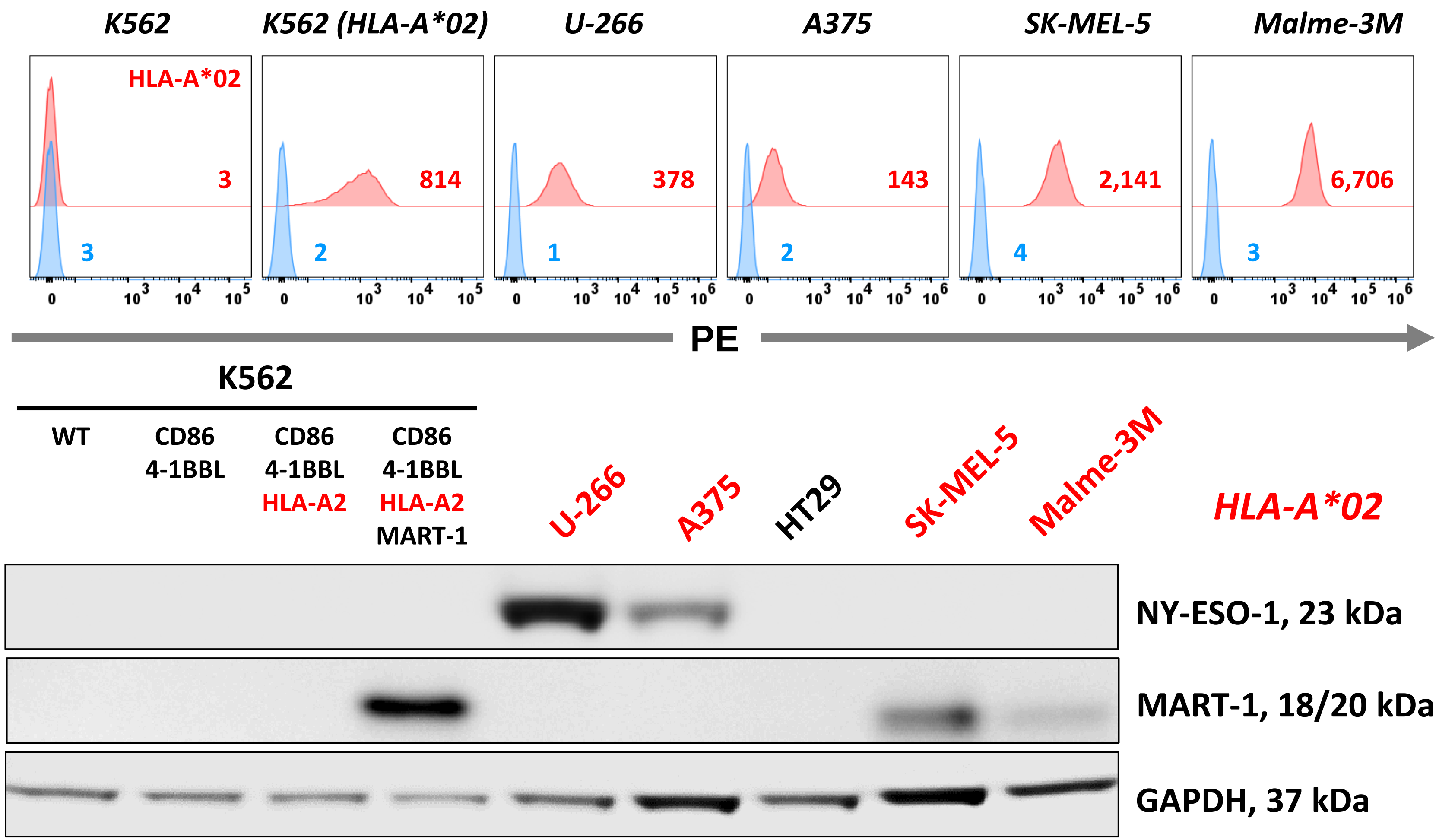

Figure S3: **Tumor cell lines expressing HLA-A*02, MART-1, and/or NY-ESO-1.** Upper panel, expression of HLA-A*02 by the indicated tumor cell lines and K562-derived aAPCS detected by flow cytometry. Blue and red numbers indicate the gMFI values of control and tested antibodies, respectively. Lower panel, expression of NY-ESO-1 and MART-1 antigens by the indicated tumor cell lines or K562-derived aAPCS detected by western blotting. Data are representative of at least two independent experiments.

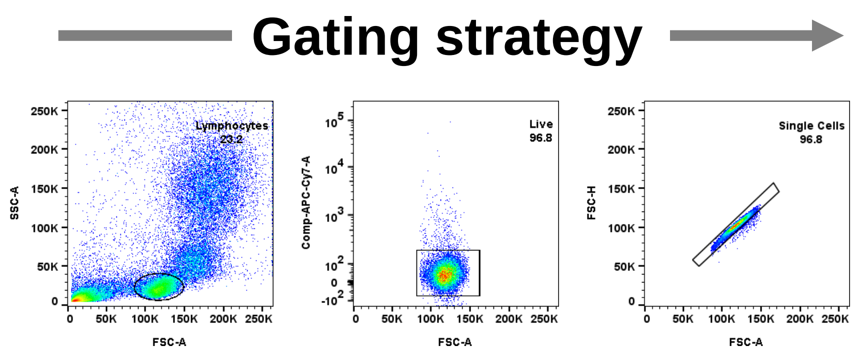

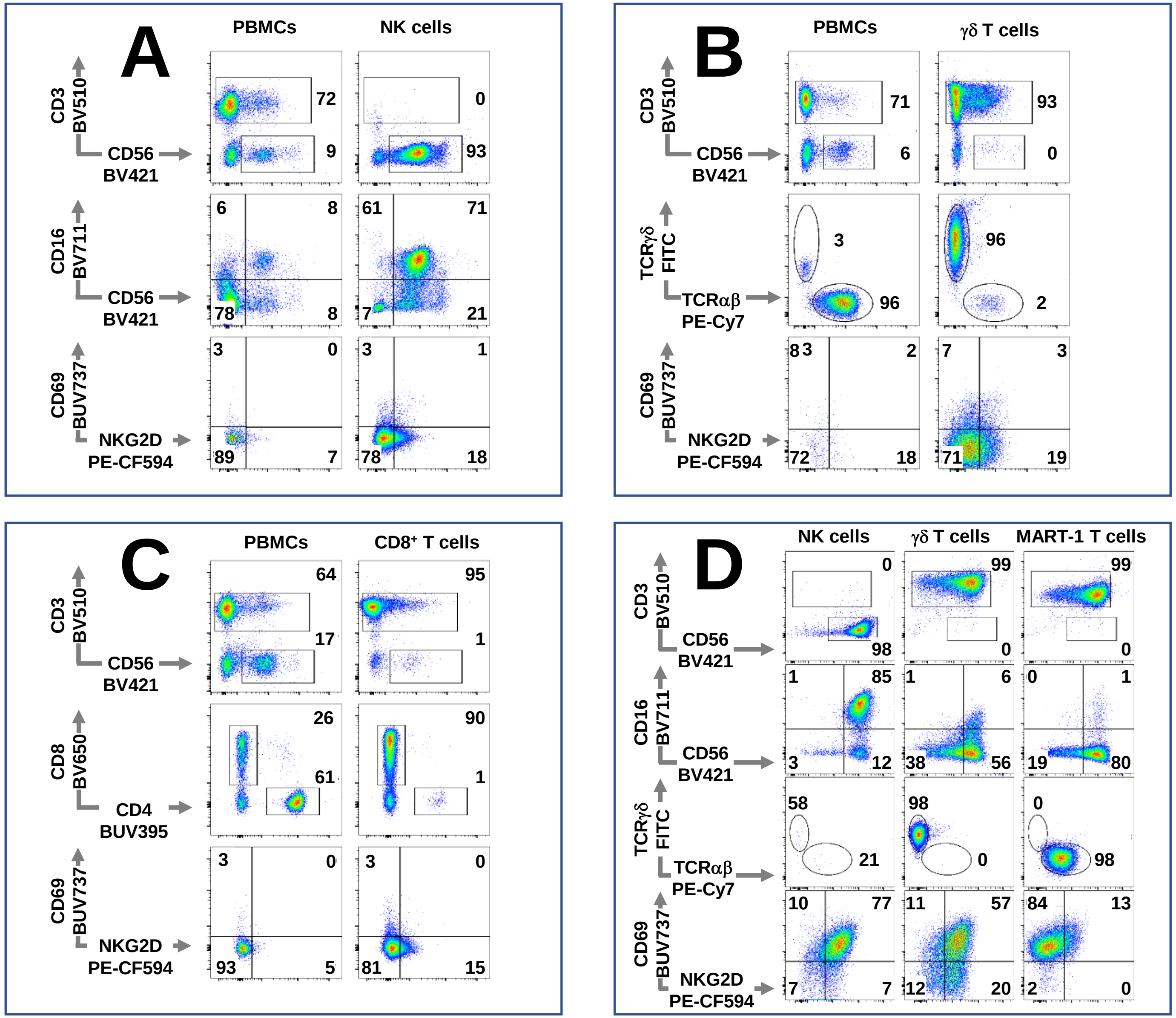

Figure S4:

Phenotype of resting (A, B, C) and effector (D) NK, γδ T, and CD8$^+$ T cells. The gating strategy is displayed above. **(A)** Purified NK cells are CD3$^-$ and CD56$^+$. **(B)** Purified γδ T cells are CD3$^+$ and TCRγδ$^+$. **(C)** Purified CD8$^+$ T cells are CD3$^+$ and CD8$^+$. **(D)** Effector NK, γδ T and CD8$^+$ T cells (HLA-A*02-restricted MART-1-specific). Data are representative of at least five independent flow cytometry experiments.

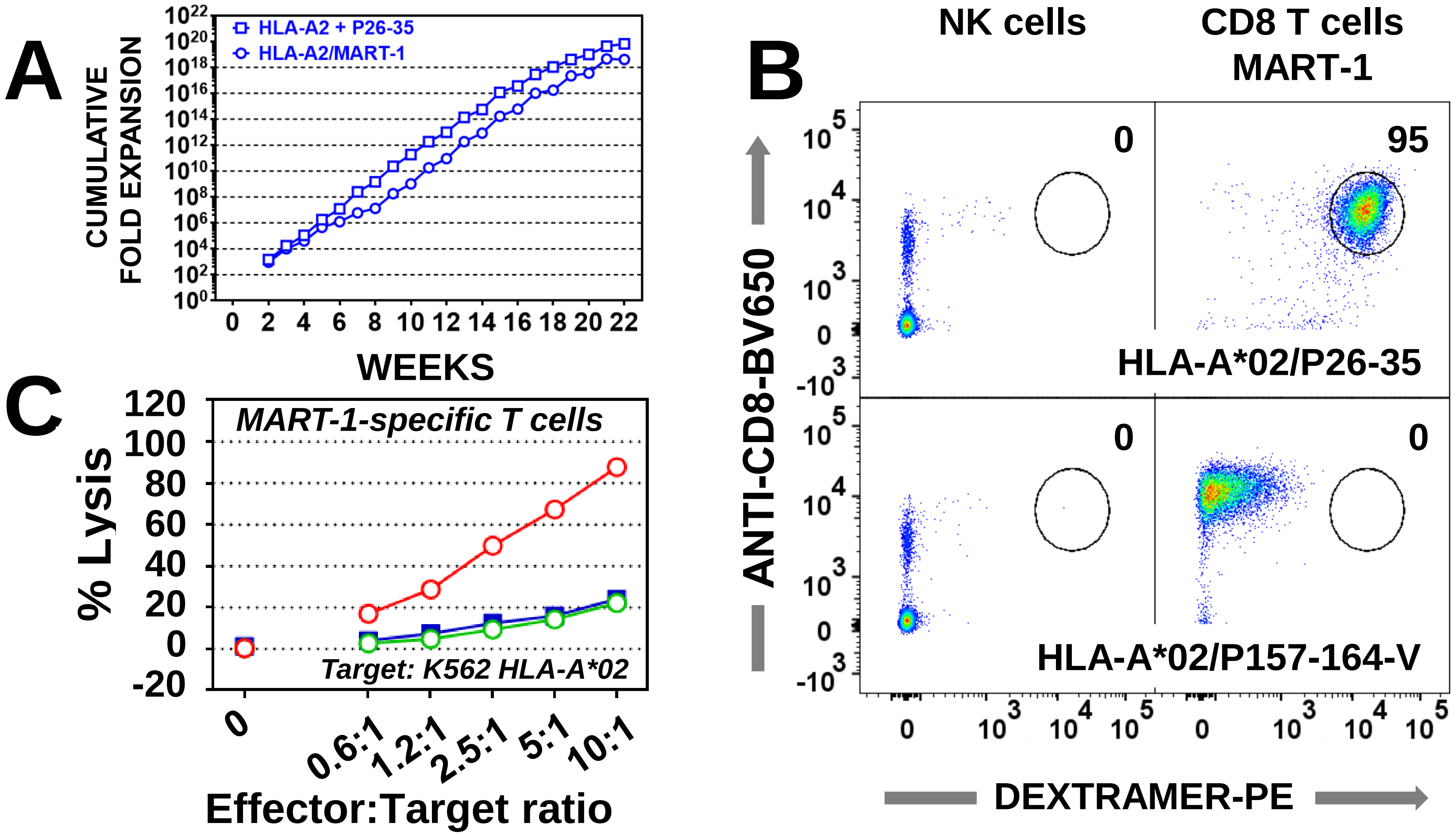

Figure S5: **Characterization of a MART-1-specific HLA-A*02-restricted CD8⁺ T cell line. (A)** The T cell line was maintained in culture for 22 weeks in the presence of cytokines (IL-7 + IL-15 + IL-21) and either K562 CD86/4-1BBL/HLA-A*02/MART-1 (open blue circles) or K562 CD86/4-1BBL/HLA-A*02 + 3 µg/mL of peptide P26-35 (open blue squares). **(B)** Effector NK cells and the CD8⁺ T cell line were stained with an anti-CD8b antibody, and either HLA-A*02/P26-35 (MART-1) or HLA-A*02/P157-164-V (NY-ESO-1) dextramer. Data are from a representative experiment repeated six times with similar results. **(C)** The cytotoxicity of the CD8⁺ T cell line against the K562 HLA-A*02 target in the absence (solid blue squares) or presence of 500 ng/mL of peptide P26-35 (open red circles) or P157-164-V (open green circles) in a 4-h calcein release assay at different E:T ratios in 96-well plates in duplicate. Data are from a representative experiment that was repeated four times with similar results.

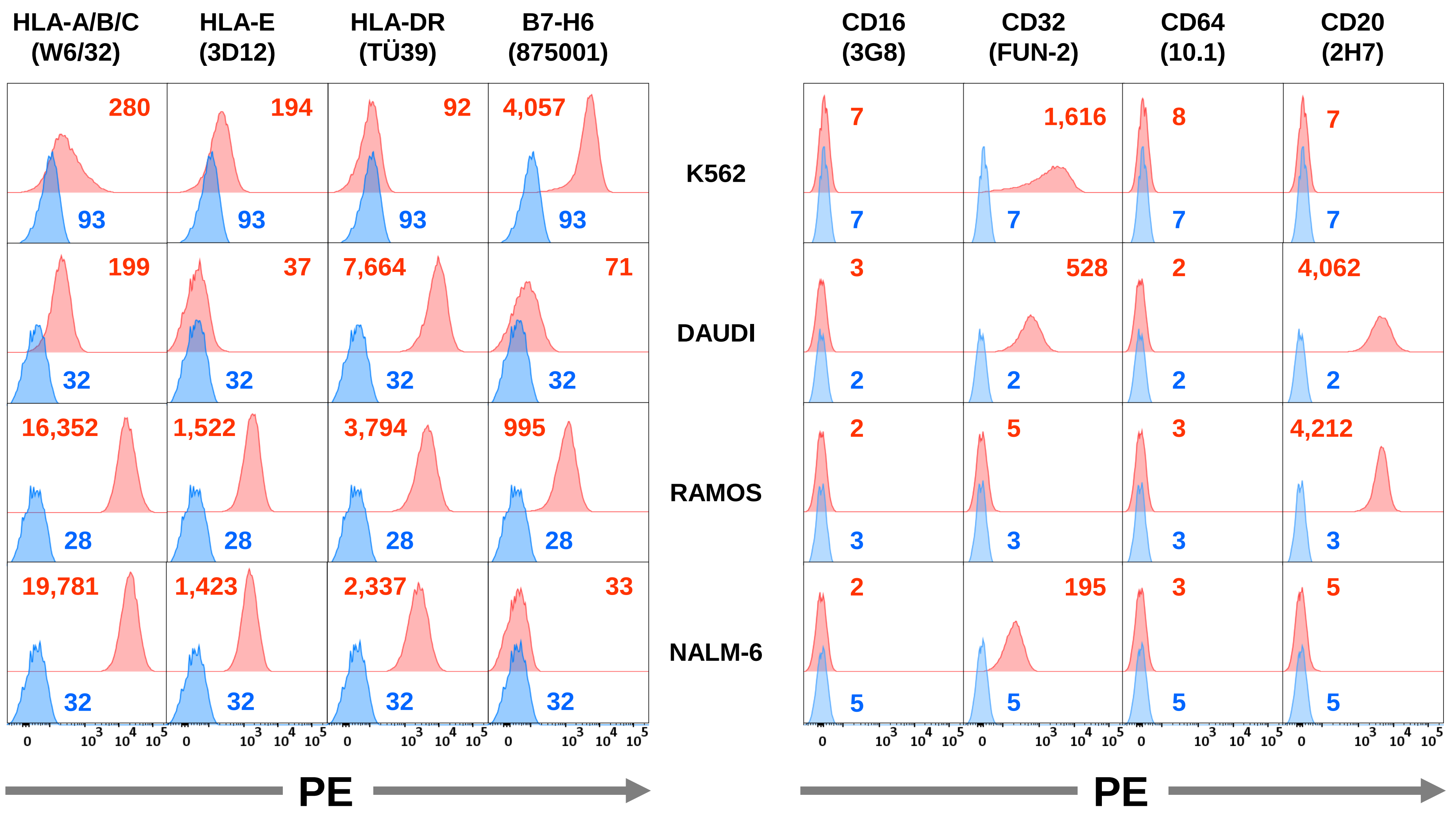

Figure S6: **Phenotype of K562, DAUDI, RAMOS and NALM-6 tumor cell lines.** Left panel, expression of HLA-A/B/C, HLA-E, HLA-DR and B7-H6. Right panel, expression of CD16, CD32, CD64 and CD20. Blue and red numbers indicate the gMFI values of control and tested antibodies, respectively. Data are representative of at least three independent flow cytometry experiments.
